# Evaluation of the Effects of Local Application of Thyme Honey in Open Cutaneous Wound Healing

**Published:** 2017-04

**Authors:** Nasrin TAKZAREE, Gholamreza HASSANZADEH, Mohammad Reza ROUINI, Azadeh MANAYI, Abbas HADJIAKHONDI, Masoumeh MAJIDI ZOLBIN

**Affiliations:** 1. Dept. of Anatomy & Histology, School of Medicine, Tehran University of Medical Sciences, Tehran, Iran; 2. Dept. of Anatomy, School of Medicine, Tehran University of Medical Sciences, Tehran, Iran; 3. Medicinal Plants Research Center, School of Pharmacy, Tehran University of Medical Sciences, Tehran, Iran; 4. Research Fellowship, Yale School of Medicine, New Haven, USA

**Keywords:** Open cutaneous wound, Honey, Wound healing, Rat

## Abstract

**Background:**

Clinicians have been searching for ways to obtain “super normal” wound healing. Honey is a traditional remedy for the treatment of infected wounds. We aimed to evaluate the wound contraction and antibacterial properties of locally produced Thyme honey on managing full-thickness wounds in vivo.

**Methods:**

This experimental study was conducted in 2015, in Department of Pharmacology, School of Pharmacy, Tehran University of Medical Sciences, Tehran, Iran on 54 adult male Wistar rats weighing 200–250 gr, and ages of 3–4 months. A square 1.5*1.5 wound was made on the back of the neck. The rats were divided into control and two experimental groups. Additionally, the control and experimental groups were separated into three subgroups corresponding to 4, 7, and 14 d of study. The control group did not receive any treatment. For histological studies, samples were taken from the wound and adjacent skin. This tissue was examined using histological staining (H&E). Wound surface and wound healing were evaluated. Data were analyzed by using one-way ANOVA with post hoc Tukey test and (*P<*0.05) was significant.

**Results:**

The macroscopic and microscopic evaluations showed that the percentage of wound healing on different days in the control and experimental groups were significant (*P*< 0.05).

**Conclusion:**

Using honey twice a day on open wounds will accelerate the healing process.

## Introduction

Wound healing is a process followed by cutaneous lesions. One of the medical science objectives is attempting to heal a wound in a shorter time span, with fewer side effects. From the long past times with development of effective methods, physicians have been seeking ways to heal wounds in the shortest possible time with the least side effects (1, 2). Several studies have been completed in connection with wound healing, using different chemicals at times and even introducing herbal materials as a catalyst, however, none have been recommended as an effective and definitive treatment (3, 4).

Wound healing is a complex and systematic process, in terms of histopathology it includes three phases including inflammation, proliferation, and remodeling. Wound healing is affected by several factors including nutrition, vitamins, hormones, oxygen, and environmental factors (5, 6). Over the course of history, natural compounds and herbal drugs were the typical bases used. In some cases, they were considered a treatment and were often packaged, marketed and distributed through the pharmaceutical industry (7–9). Presently, a trend has resurfaced that encourages the use of herbs and natural ingredients because of their minimal side-effects, also, WHO have been recommending these types application (10, 11). Honey has long been determined from thousands of years ago to treat infected wounds (11, 12). Its antimicrobial effects (inhibitory) demonstrate on many types of aerobic bacteria, anaerobic, gram-positive and gram-negative (13, 14). High concentrations of glucose and carbohydrates in honey have inhibitory effect on the growth of microbial agents (13, 15, 16).

Injury caused vascular system damage subsequently hypoxia and lactate increase occurred, then these area’s cells sent signals, which resulted in angiogenesis via increasing fibroblasts and connective tissue and granulation tissue. The concentration of hydrogen peroxide in honey is 1 milmol/L, released slowly in wound bed and plays important role in the elimination of microbial agents. Hydrogen peroxide with its insulin-like effect, influence the cells involved in the healing and will cause to angiogenesis (17–20). Wound healing is a dynamic response to injury and requires interactions between different cell types, structural proteins and growth factors. Macrophages, neutrophils, and fibroblasts cells have important role in wound healing. Newly, proliferated fibroblasts secrete connective tissues elements like collagen fibers and proteoglycan that is leading to restoration of wound edges toward together. Fibroblasts cells are an important part of wound repair because as cells proliferate in the wound, collagen emerges resulting in faster wound healing (21, 22). Acidic pH of honey along with its osmotic effects, would stimulate the activity of phagocytes and lymphocytes in the wound and increase the other antibacterial components (23, 24). Proline in honey with antioxidant activity prevents the formation of free radicals and it is responsible for anti-inflammatory effects of honey (25, 26). Combinations of various properties of honey enhanced angiogenesis, granulation, and epithelialization and finally accelerate the wound healing (27, 28).

According to the general characteristics of honey and the lack of an effective drug for the treatment of wounds, this study aimed to evaluate the effect of local application of honey and the number of effective usage of it on open cutaneous wound healing in rats, all these procedures were evaluated by cell counting.

## Materials and Methods

### Animals

This experimental study was conducted in 2015, in Department of Pharmacology, School of Pharmacy, Tehran University of Medical Sciences, Tehran, Iran on 54 adult male Wistar rats weighing 200–250 gr, and ages of 3–4 months. They were placed in individual cages during the study period, 12 h of darkness and 12 h of light were available, and have ready access to water and food. The rats were divided into control and two experimental groups. Additionally, the control and experimental groups were separated into three subgroups corresponding to 4, 7, and 14 d of study. In first experimental group, honey was used once on the wound. The second experimental group received honey overtreatment twice on the wound. The control group did not receive any treatment.

Animal experiments were approved by the Ethics Committee of Tehran University of Medical Sciences in accordance with the university’s guidelines (No. 56-27372).

### Surgical method

On the day of surgery, rats were anesthetized with IP injection of ketamine hydrochloride, (Ketalar, Gedeonrichter, Germany) at a dose 5 mg/100 gr of body weight. Diazepam (*Chemidarou,* Iran) was also injected, at a dose of 4.5 mg/kg of body weight as a muscle relaxant and pentazocine (Toliddaru, Iran) was used as pre-anesthetic at a dosage of 0.04 mg/100 gr hundred grams of body weight. The rats’ neck hair was shaved and disinfected with povidyn - Iodine (Chemidarou, Iran). Under sterile conditions, a 1.5× 1.5 mm incision was created on the neck of each rat spanning the full thickness of skin. Surgery day was considered day zero. After surgery, the wound washed with saline then its surface was covered with a fixed amount of honey thyme of Damavand, Tehran, Iran.

### Honey

Mono flower honey was collected from the nectar of thymus plants in late spring from the mountainous region of Damavand in Iran. For isolation of impurity, honey was passed from 0.5 mm Whatman filter at 25–30 °C temperature, high temperature is not used in any way because the high heat causes loss of useful compounds of honey, such as proline amino acid. This honey has less than 10% water and more than 90% sugar transferred within dark lid dish and used in research laboratory.

### Wound healing assay

The sizes of lesions were measured by transparent sheet on different days of wound recovery days 3, 5, 7, 9, 11, and 13. Wound recovery percentage was evaluated with following formula:

Recovery percentage = (Wound surface on the first day − wound surface on day X) * 100

Wound surface on the first day X is the surface measurement on given day.

### Histological study

After completion of treatment, animals were sacrificed by inhalation of ether. Tissue samples were collected from the full skin thickness of the entire wound area as well as surrounding normal tissues. Tissues were fixed in 10% buffered formalin and further prepared for fixation in paraffin blocks. Serial 5 mm sections were obtained and stained with H&E and specific Masson’s Trichrome to assess the density of collagen fibers. Using a light microscope (CX31-OLYMPUS, Japan) and a magnification field of 40*/0.65, ten areas on the wounds bed were investigated. Via microscopic examination, fibroblast cells, neutrophils, macrophages, and vascular sections in the wound bed were counted using Image Tools 3 software.

### Statistical Analysis

All data was entered into SPSS 16 (SPSS, Chicago, IL, USA) for statistical analyses. Quantitative data are expressed as Mean ± SD analyzed by multivariate analysis of variance (MANOVA). ANOVA with post hoc Tukey and repeated measurement was used for comparing the quantitative data. *P-*value<0.05 was considered as significant level.

## Results

**Fig. 1:**
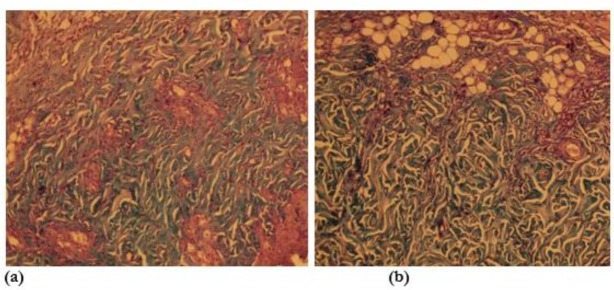
Microscopic view of open cutaneous wounds: **(a)** control group, collagen fibers are lesser than the experimental honey twice daily group in this photomicrograph. **(b)** Experimental honey twice daily group, collagen fibers are more than the control group in this photomicrograph (specific staining, Masson’s trichrome *10)

Wound healing process was assessed by microscopic and macroscopic study. In macroscopic study, lesions size was measured on different days (Fig. 1–b). In comparison, between lesion surface and percentage of wound healing, there was a significant difference between control and experimental groups (Fig. 1a, b).

There was no significant difference in wound surface on the first day after surgery. However, by the third day of treatment with Honey, the wound area significantly was decreased, in comparison with the control and experimental groups (*P*<0.05). In the experimental groups, wound healing from the third day to the fourteenth day of treatment with Honey was significantly greater by percentage than the control groups (*P*<0.05).

**Fig. 2:**
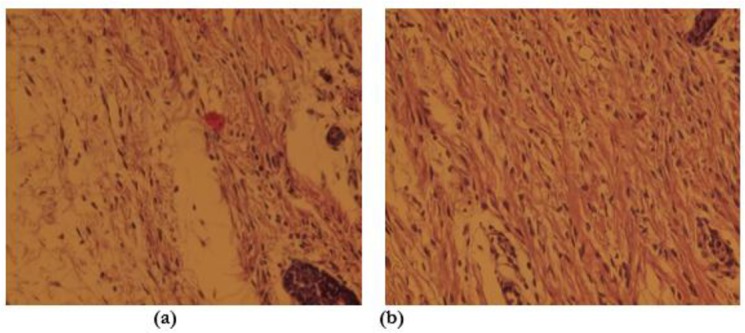
Microscopic view of open cutaneous wounds: **(a)** Control group, the number of Fibroblast are lesser than the experimental honey twice daily group in this photomicrograph. **(b)** Experimental honey twice-daily group, the number of Fibroblast is more than the control group in this photomicrograph. (Staining, H&E *40)

In microscopic examination of the samples in both Massons’ trichrome and H&E staining, the number of fibroblasts, macrophages, neutrophils and collagen fibers in the experimental groups and control group had significant difference (*P*<0.001) (Table 1). The average and standard deviation of the number of fibroblasts, macrophages, neutrophils and collagen fibers in the experimental groups and control group during the fourth, seventh and fourteenth days in different experimental groups were recorded in Table 1. In the experimental honey twice-daily group, collagen fibers were more than the control group (Fig. 1). In the experimental honey twice-daily group, the numbers of fibroblast were more than the control group (Fig. 2).

**Table 1:** Comparing the macrophages, fibroblasts, neutrophils and vessels number in the wound bed of groups in the 4^th^, 7^th^ and 14^th^ day of study

	**4th Day**						**7th Day**						**14th Day**					

	**Groups**						**Groups**						**Groups**					
	**Control**		**1^st^ Experiment**		**2^nd^ Experiment**		**Control**		**1^st^ Experiment**		**2^nd^ Experiment**		**Control**		**1^st^ Experiment**		**2^nd^ Experiment**	
	Mean (N*)	SD**	**Mean (N)**	**SD**	**Mean (N)**	**SD**	**Mean (N)**	**SD**	**Mean (N)**	**SD**	**Mean (N)**	**SD**	**Mean (N)**	**SD**	**Mean (N)**	**SD**	**Mean (N)**	**SD**
Macrophages	6.20	.80	4.60 ■	.40	3.40 ■	.51	7.80	.66	5.6■	.24	4.0■	.32	1.40	.40	.80	.37	.40	.24
Fibroblasts	89.6	2.77	105■	1.9	123■	1.00	125	3.02	125■	3.09	152■	1.75	136	1.58	160■	1.1	182■	1.16
Neutrophils	16.2	.66	14■	.71	12■	.71	10.8	.58	11.4	.75	12.0	.32	7.80	.37	5.0■	.71	3.0■	.71
Vessels	5.20	.58	5.80	.58	10.00	2.55	10.6	.75	12.6	.40	12.2	1.02	12.40	1.44	13.0	1.4	14.8	.97

■ Significant differences between experimental groups and control group (*P*≤0.05)

* Number /

** Standard Deviation

## Discussion

Wound healing is a process that occurs after skin injury, is a dynamic response to injury, and requires interactions between different cell types, structural proteins and growth factors. Macrophages, neutrophils, and fibroblasts cells have important role in wound healing. Wound healing is divided into three phases of inflammation, proliferation, and remodeling (reconstruction). Immediately after a skin ulcer cell reaction occurs, initially blood clotting and degranulation of mast cells happens. Then chemical mediators are released and finally inflammation phase occurs (5, 6). Blood clots are the initiator of recovery signals, in this process, Thrombin attracts macrophages to the damaged tissue, platelets, and fibrin stimulates macrophages to secrete wound regenerative signals. In inflammatory phase, which begins a few hours after injury, primarily neutrophils followed by macrophages, reach to maximum amount (25). Honey contains moisture absorption properties that can reduce edema of the wound, resulting in faster healing and early proliferation phase of inflammation process (23–25).

The seventh day of this study, considered as the proliferative phase of wound healing process. Proliferation phase begins after the inflammatory phase and fibroblasts are activated in the wound area. Angiogenesis or vascularization is a necessary element of repair, with adding chemotactic factors to endothelial cells; angiogenesis is inducible. Hydrogen peroxide produced by honey with its insulin-like properties, stimulate cell proliferation and angiogenesis in wound bed furthermore (Table 1) it has an important role in the elimination of microbial agents (17, 18, 25). Lactate synthesis and released mediators from macrophages lead to fibroblasts proliferation and new blood vessels formation (15). Angiogenesis is an important factor in wound healing process, blood vessels leading to nutrition, oxygenation, cell proliferation and ultimately accelerate wound healing (26, 27). Growth of blood vessels in the experimental group in comparison with control group had an Increasing trend (Table 1). Honey can accelerate angiogenesis and granulation tissue formation in wound area (22, 29). In this study, the number of Fibroblasts was significantly (*P*<0.05) higher in the experimental group (Table 1).

Fibroblasts in response to physiological and pathological stimuli, proliferate and can re-create the original tissue and repair wound. Honey through increasing angiogenesis make nutrient and oxygen available to fibroblasts, and its acidic pH release oxygen from hemoglobin and leading to increased activity of fibroblasts and collagen formation is accelerated (Fig. 1) (21, 23).

**Fig. 3:**
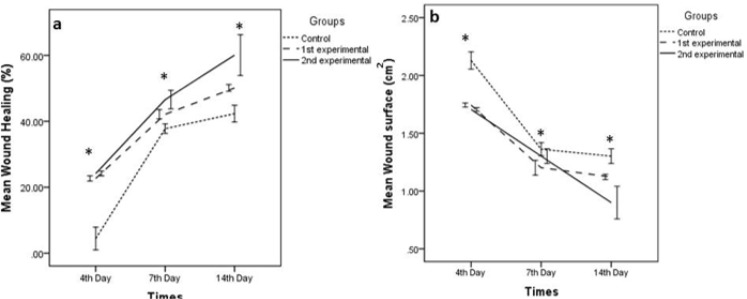
**a)** Comparing the wound healing percentage between control and experimental groups in the different days of study. **b)** Comparing the wound surface between control and experimental groups in the different days of study/*Significant differences between experimental groups and control group (*P*≤0.05)

A significant increase of fibroblast cells in the experimental groups showed a positive effect of honey on proliferative phase of wound healing process (22) (Table 1). Fibroblasts were synthesized part of the extracellular matrix, such as fibronectin and proteoglycans that they provide suitable migration and proliferation of cells (22, 27). Fibroblasts are then synthesized collagen, the proliferation of fibroblasts in the group treated with honey was increased as compared with control group, similarly results we found in this research (27). In the review of the fourth day’s samples, the numbers of fibroblasts in second experimental group in comparison with control group indicate that the proliferative phase begins earlier in experimental group while the control group still in the inflammatory phases (Table 1). Newly proliferated fibroblasts (Fig. 2) secrete connective tissues elements like collagen fibers and proteoglycan that is leading to restoration of wound edges toward together. Increased numbers of fibroblasts will result to collagen increasing caused to reduce the size of the wound (Fig. 3).

An accumulation of lesions and reduction of their size are related to fibroblasts and myofibroblasts activity in the granulation tissue (Table 1). Epithelialization and differentiation of epidermis continue at the maximum rate as long as the wound is kept moist. Percent and the rate of wound healing in the experimental group received honey twice a day had significantly higher (Fig. 1). Percent and the rate of wound healing in second experimental group that received honey twice a day were significantly higher (Fig. 3).

## Conclusion

Local application of honey twice daily enhanced the healing process, shortening the inflammatory phase, an increase of granulation tissue, angiogenesis and early proliferative phase and remodeling and finally wound heal faster.

## Ethical considerations

Ethical issues (Including plagiarism, informed consent, misconduct, data fabrication and /or falsification, double publication and /or submission, redundancy, etc.) have been completely observed by the authors.
